# Early Resolution of Abnormal Vascular Networks After Superficial Temporal Artery to Middle Cerebral Artery (STA-MCA) Bypass Surgery for Twig-Like Middle Cerebral Artery With Intracerebral Hemorrhage: A Case Report

**DOI:** 10.7759/cureus.72740

**Published:** 2024-10-30

**Authors:** Astushi Tsukada, Koji Hirata, Kyoji Tsuda, Keishi Fujita, Kiyoyuki Yanaka, Eiichi Ishikawa

**Affiliations:** 1 Department of Neurosurgery, Ibaraki Seinan Medical Center Hospital, Sashima, JPN; 2 Neurosurgery, University of Tsukuba Hospital, Tsukuba, JPN; 3 Neurosurgery, Tsukuba Memorial Hospital, Tsukuba, JPN

**Keywords:** encephalo-duro-synangiosis, intracerebral hemorrhage, moyamoya disease, sta-mca bypass, twig-like middle cerebral artery

## Abstract

The twig-like middle cerebral artery (T-MCA) is a rare condition characterized by unilateral middle cerebral artery (MCA) occlusion and abnormal vascular networks, resembling Moyamoya Disease (MMD). While some reports mention bypass surgery for T-MCA, akin to MMD, there is no established treatment method. Previous reports highlight the efficacy of revascularization procedures in preventing recurrent strokes in T-MCA patients, but there is limited data on its usefulness for cases with intracerebral hemorrhagic onset. We present the case of a 37-year-old man in whom bypass surgery for T-MCA with intracerebral hemorrhage was successful, and the periventricular anastomosis, which could have caused the hemorrhage, was resolved early. The patient showed improvement after surgery, and the subsequent MRI, seven days after surgery, confirmed the disappearance of the periventricular anastomosis. Remarkably, no stroke recurrence was observed throughout the 15-month follow-up. This case suggests the usefulness of bypass surgery for T-MCA with hemorrhagic onset, especially when periventricular anastomosis is involved. Further research to determine the optimal timing and treatment approaches for such cases is required.

## Introduction

Twig-like middle cerebral artery (T-MCA) is a rare anomaly characterized by unilateral MCA occlusion with an abnormal vascular network [1-3]. This condition shares similarities with Moyamoya disease (MMD), and although there have been occasional reports of bypass surgery, there is no established treatment method [1-8]. In any case, conservative treatment was performed. However, we demonstrated the effectiveness of revascularization procedures in preventing recurrent strokes in patients with T-MCA [4,5]. However, little is known about the usefulness of bypass surgery for T-MCA with intracerebral hemorrhagic onset, particularly regarding the timing of the periventricular anastomosis resolution, which is a potential risk factor for bleeding in MMD [6]. In this case, we encountered a patient with T-MCA and intracerebral hemorrhage (ICH) who underwent bypass surgery. The bleeding was attributed to periventricular anastomosis, which resolved shortly after the surgery. This case points to the potential efficacy of revascularization in managing T-MCA with intracerebral hemorrhagic onset.

## Case presentation

**Figure 1:**
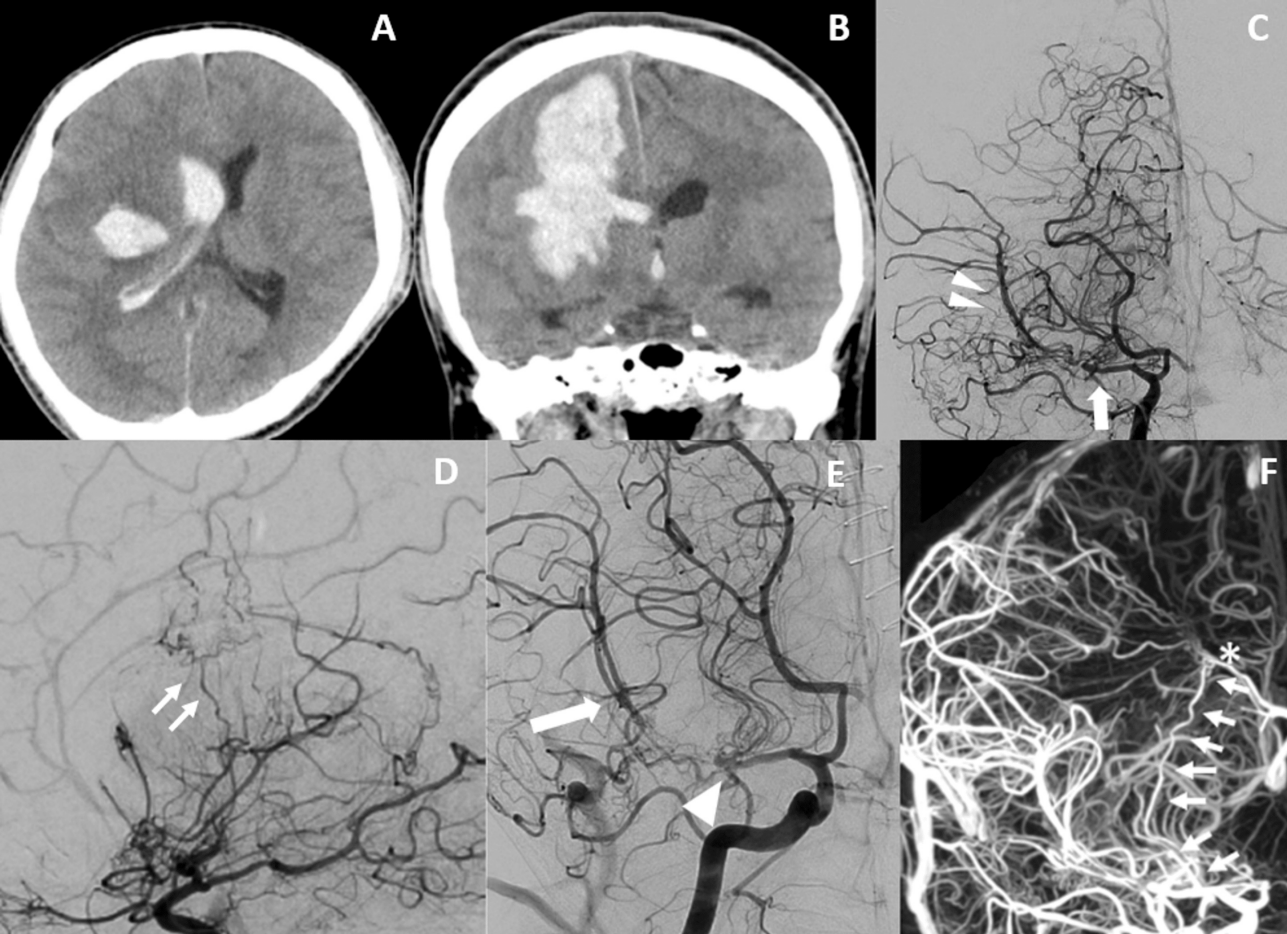
CT and artery angiography on admission A, B: Head CT on admission (A: axial B: coronal). About 66 ml of cerebral hemorrhage from the right putamen to the frontoparietal cortex and　hydrocephalus due to perforation into the lateral cerebral ventricle. C-F: Right internal carotid artery angiography (C: anterior-posterior view D: lateral view E: anterior-posterior view capturing the characteristics of T-MCA F: Vaso CT). Vascular occlusion in the right MCA M1 portion and a vascular network in the surrounding area (C: arrow). There was no narrowing of the terminal segment of the internal carotid artery and distal MCA to the steno-occlusive lesion (C: double arrowhead, E: arrowhead). Periventricular anastomosis was observed between the dilated LSA (F: arrow) and the medullary artery (E: double arrow F: arrow, asterisk).

A 37-year-old man was brought to our hospital due to sudden consciousness disturbance and left-sided weakness. He had untreated hypertension, and upon admission, his blood pressure was high at 186/62 mmHg. He had a Glasgow Coma Scale (GCS) score of 14 and exhibited severe left-sided weakness. Head CT revealed an intracerebral hemorrhage extending from the right putamen to the frontoparietal cortex, along with mild ventricular rupture (Figures 1A, 1B). Despite motion artifacts hindering precise evaluation, no apparent vascular abnormalities, such as cerebral aneurysm or arteriovenous malformation, were detected on contrast-enhanced CT. Within three hours, his level of consciousness worsened to a GCS score of 11. Follow-up CT scan revealed increased hematoma. Emergency craniotomy was performed to evacuate the hematoma, although the exact source of the bleeding remained elusive.

CT scan performed the next day confirmed that the hematoma had been completely removed. Digital subtraction angiography (DSA) to investigate the source of the hemorrhage revealed occlusion of the M1 segment of the right middle cerebral artery with a twig-like network of abnormal vessels in its vicinity, but no stenosis in the MCA distal to the occlusion was observed. There was no leptomeningeal anastomosis, but periventricular anastomosis was observed between the dilated lenticulostriate artery (LSA) and the medullary artery (Figures 1D-1F).

**Figure 2:**
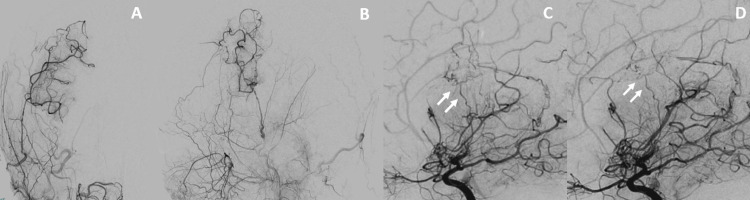
Artery angiography after bypass surgery A, B: Right external carotid artery angiography after bypass surgery (A: anterior-posterior view B: lateral view). Efficient internalization from the superficial temporal artery was confirmed. C, D: Comparison of right internal carotid artery angiography before(C) and after(D) bypass surgery. The double arrow showed shrinkage of periventricular anastomosis.

**Figure 3:**
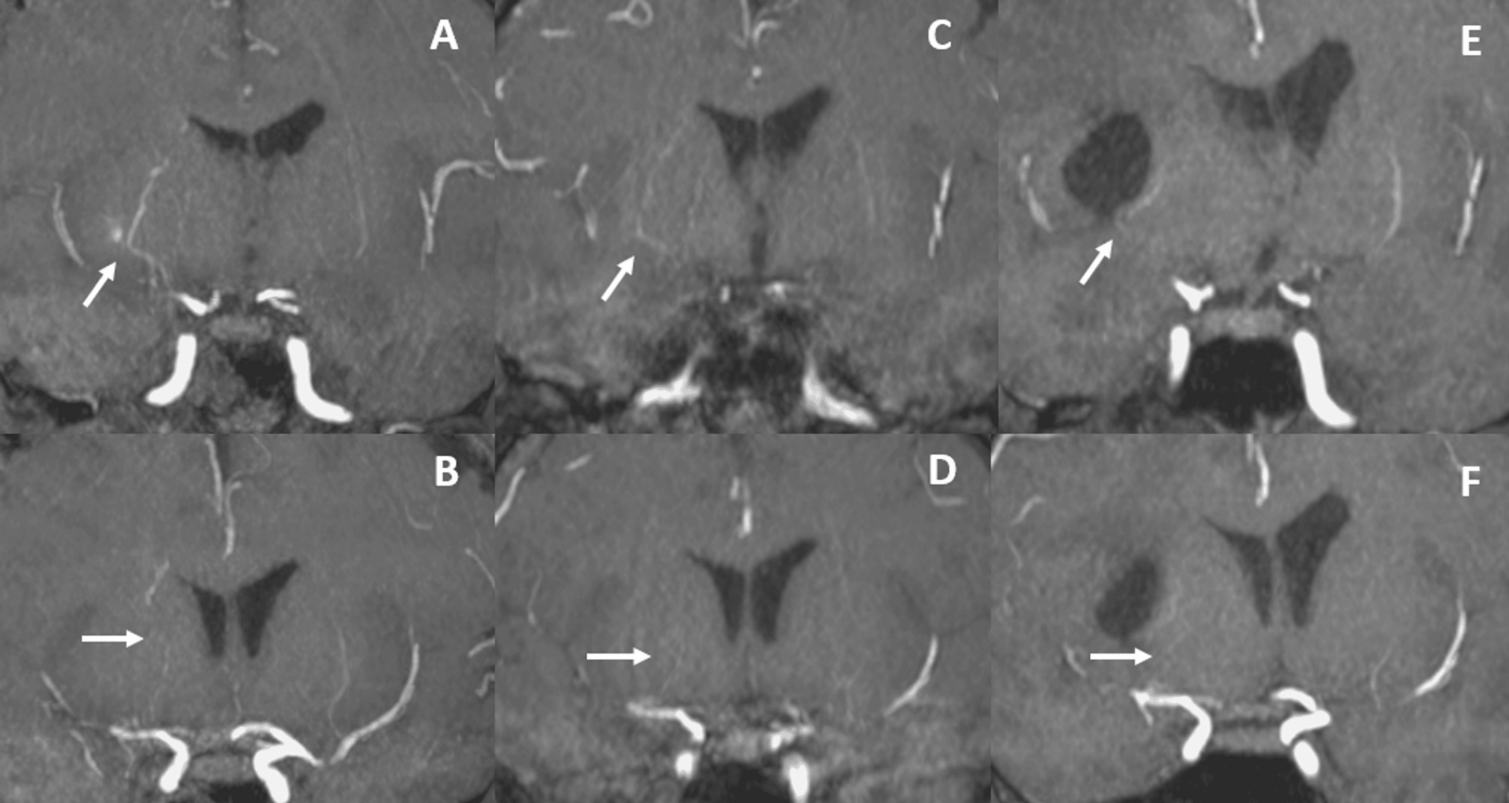
Comparison of MRI, 1 mm slice of Maximum Intensity Projection (MIP) before and after bypass surgery (A, B: Magnetic resonance angiography (MRA) before bypass surgery C, D: MRA seven days after bypass surgery E, F: MRA seven months after bypass surgery). MRI shows shrinkage of periventricular anastomosis (arrow).

A superficial temporal artery to middle cerebral artery (STA-MCA) bypass and encephalo-duro-synangiosis (EDS) were performed to prevent rebleeding (Figure 2). A vessel of the cortical portion of the MCA (M4) on the brain surface, perfused by the periventricular anastomosis, was selected as the recipient for the STA-MCA bypass. There were no major complications during the perioperative period. On the seventh and 12th days after the operation, MRI (Figure 3) and DSA were performed, confirming the bypass vessel’s patency and the shrinkage of the periventricular anastomosis (Figure 2D, 3B). Transdural anastomosis through indirect bypass was not observed (Figure 2A, 2B).

The patient was transferred to a rehabilitation hospital three weeks after surgery. Seven months later, magnetic resonance angiography showed further shrinkage of the periventricular anastomosis (Figure 3C). Although he remained partially paralyzed, he was able to reintegrate into society using a lower limb assistive device. Over the 15-month follow-up period after the bypass surgery, no recurrent strokes were observed. Given our incomplete understanding of the pathology of this disease, we decided to continue regular MRI follow-ups.

## Discussion

T-MCA is characterized by an abnormal plexiform arterial network with multiple channels, known as a twig-like network (TLN). The periventricular vascular network is usually not prominent in T-MCA [8]. The lenticulostriate arteries (LSAs) arise from the TLN, and distal branches of the MCA beyond the TLN maintain nearly normal vessel caliber with anterograde blood flow. The TLN is supplied by the steno-occlusive MCA or anomalous arteries originating from various segments of the anterior cerebral artery (ACA), anterior choroidal artery, or internal carotid artery terminus. All TLNs join the distal M1 or proximal M2 segment of the MCA. The periventricular vascular network is usually not prominent in T-MCA [1-5]. In contrast, MMD is a progressive steno-occlusive vasculopathy affecting the internal carotid artery bifurcation and its major branches, with the secondary formation of an extensive collateral network at the base of the brain, known as moyamoya vessels.

The exact cause of intracerebral hemorrhage (ICH) associated with T-MCA remains unclear, but three potential causes have been proposed: 1) TLN vulnerability around the MCA occlusion; 2) TLN vulnerability around the periventricular anastomosis; or 3) rupture of an aneurysm within or outside the TNL [4,5]. In our case, the absence of aneurysms intraoperatively and the presence of hematoma along the periventricular anastomosis on radiological images suggested that the vulnerability of the periventricular vascular network was considered to cause cerebral hemorrhage. Since the periventricular anastomosis disappeared after bypass surgery, effective prevention of rebleeding can be expected.

Twelve cases of bypass surgery for hemorrhagic onset T-MCA, including our previous cases, were reported (Table 1) [2,4,9-15], with a mean age of 51.4 years, including 10 cases of intracerebral hemorrhage, two cases of subarachnoid hemorrhage, and three cases of intraventricular hemorrhage, higher prevalence in Asian populations [2,4,9-15]. Abnormal vessel network shrinkage was observed in five of the eight patients. Surprisingly, in the present case, the periventricular anastomosis began to disappear only seven days postoperatively. It may help prevent postoperative secondary stroke by reducing hemodynamic stress. In pediatric MMD, neovascularization is observed three to six months after indirect revascularization [16-18], but the effect is less pronounced in adults than in children with greater potential for angiogenesis. Since most patients with T-MCA are adults, the potential for angiogenesis is small, and direct revascularization seems to be the preferred method over indirect revascularization. Compared to MMD, there is no narrowing of the recipient blood vessels, so a special STA-MCA bypass is not required in T-MCA.

**Table 1:** Summary of cases performed bypass surgery due to intracranial hemorrhage in T-MCA T-MCA: twig-like middle cerebral artery, ICH: intracranial hematoma, SAH: subarachnoid hemorrhage, IVH: intraventricular hemorrhage, NA: not available, AN: abnormal network, PA: periventricular anastomosis, D: day M: month Y: year.

Author Year		Age/Sex	Clinical presentation		treatment		onset to treatment		Follow-up imaging modalities			outcome			Treatment to shrinkage	
Seo et al., 2012 [2]		49/F	ICH		EDAS		NA		DSA			No change			NA	
Takarada et al., 2021 [4]		46/F	IVH		STA-MCA bypass Clipping		NA		DSA			No change			6M	
Goto et al., 2019 [9]		51/M	ICH, IVH		STA-MCA bypass		NA		NA			NA			NA	
Rodriguez et al., 2011 [10]		52/M	ICH		STA-MCA bypass		NA		NA			NA			NA	
Seno et al., 2017 [12]		49/F	SAH, IVH		STA-MCA bypass		2D		MRI			Shrinkage of AN			1Y	
Inoue et al., 2016 [11]		55/M	ICH		STA-MCA bypass Clipping		6M		DSA			Shrinkage of AN			1Y	
Hirai et al., 2018 [13]		63/F	ICH		STA-MCA bypass		25D		NA			NA			NA	
Hirai et al., 2018 [13]		73/F	SAH, ICH		STA-MCA bypass		26D		NA			NA			NA	
Yamada et al., 2018 [14]		68/M	ICH, CI		STA-MCA bypass		180D		MRI			Shrinkage of AN Shrinkage of PA			6M	
Fuse et al., 2021 [15]		28/F	ICH		STA-MCA bypass		3M		MRI			Shrinkage of AN Shrinkage of PA			3M	
Fuse et al., 2021 [15]		46/F	ICH		STA-MCA bypass		NA		MRI			Shrinkage of AN Shrinkage of PN			2M	
Present case		37/M	ICH		STA-MCA bypass EDAS		1M		MRI, DSA			Shrinkage of PN			7D	

The limitation of our case is that it is a single report, so we do not know whether our treatment approach and the follow-up period are appropriate. However, the patient showed the beginning of the abnormal vascular network regression on postoperative day 7, suggesting that early direct revascularization is desirable. Because brain swelling and conditions unfavorable to anastomosis can be expected immediately after hemorrhage, we suggest that the timing of surgery should be determined on a case-by-case basis, with the principle of performing surgery as early as possible.

We recently reported a new case of acquired T-MCA, suggesting that T-MCA may be a secondary sequela, similar to some cases of MMD [19]. T-MCA and MMD may be considered diseases related to the same gene; thus, treatment methods for MMD should be considered in patients with T-MCA. While there are many pediatric cases of MMD, most cases of T-MCA occur in adults, with very few pediatric cases. In other words, individuals with T-MCA may remain asymptomatic until adulthood, and other factors, such as atherosclerotic changes, could also be involved in the onset of T-MCA symptoms [5]. Therefore, when considering treatment strategies, it is necessary to consider the potential for angiogenesis depending on age. More cases need to be accumulated to establish the optimal treatment and timing.

## Conclusions

The clinical outcomes of the present case suggest that, with prompt intervention, STA-MCA bypass can be used to effectively manage T-MCA with intracerebral hemorrhage, indicating its potential for preventing rebleeding and early disappearance of abnormal vascular networks. Further research on the optimal timing for surgery is warranted for long-term prevention and long-term follow-up is crucial to understanding T-MCA pathology and ensuring effective prevention of rebleeding.
